# Dasatinib has a dual effect on sepsis

**DOI:** 10.1186/cc13008

**Published:** 2013-11-05

**Authors:** Cassiano F Gonçalves-deAlbuquerque, Alessandra F Silveira, Cristina L Nagae, Carlos André M Silva, Mirian Priscila L Lima, Raysa Captivo, Larissa Camisão, Caroline L Hildebrandt, Patrícia T Bozza, Adriana R Silva, Hugo C Castro-Faria-Neto

**Affiliations:** 1 Laboratório de Imunofarmacologia, IOC/Fiocruz, Rio de Janeiro, Brazil

## Background

Sepsis occurs as a result of a systemic inflammatory response to an infection. In this context, homeostasis of biological systems depends on regulatory mechanisms to modulate the amplitude of the immune response to stimuli, such as infection, preventing damage resulting from this imbalance of immune response. The exacerbated immune response can cause serious tissue or systemic damage, as occurs in autoimmune and chronic inflammatory diseases. The main aim of our study is to investigate the effect of dasatinib in polymicrobial sepsis.

## Materials and methods

Swiss mice were subjected to cecal ligation and puncture and treated with dasatinib 1, 5 and 10 mg/kg 30 minutes before and 6 and 24 hours after the surgery. Survival rate and clinical signs were assayed; cell accumulation, bacterial load were measured in peritoneal lavage and inflammatory mediators were measured in plasma.

## Results

Animals receiving dasatinib 5 and 10 mg/kg showed the worst clinical score and an increased mortality rate. Animals receiving dasatinib 1 mg/kg showed an increase in survival, a decrease in clinical score, in cell migration, in colony-forming units and cytokine production.

## Conclusions

Dasatinib has a dual effect in polymicrobial sepsis, where higher doses had deleterious effects but lower doses had beneficial effects, probably because lower doses may downregulate the immune response, avoiding extensive tissue damage.

